# Genome Report: Genome sequence of 1S1, a transformable and highly regenerable diploid potato for use as a model for gene editing and genetic engineering

**DOI:** 10.1093/g3journal/jkad036

**Published:** 2023-02-09

**Authors:** Thilani B Jayakody, John P Hamilton, Jacob Jensen, Samantha Sikora, Joshua C Wood, David S Douches, C Robin Buell

**Affiliations:** Department of Plant, Soil and Microbial Sciences, Michigan State University, Plant and Soil Sciences Building, 1066 Bogue Street, Room A286, East Lansing, MI 48824, USA; Center for Applied Genetic Technologies, University of Georgia, 111 Riverbend Rd, Room 249, Athens, GA 30602, USA; Department of Crop & Soil Sciences, University of Georgia, 111 Riverbend Rd, Room 249, Athens, GA 30602, USA; Department of Biochemistry, Molecular Biology, and Biophysics, University of Minnesota, Jackson Hall, 321 Church Street SE, Minneapolis, MN 55455, USA; Department of Plant, Soil and Microbial Sciences, Michigan State University, Plant and Soil Sciences Building, 1066 Bogue Street, Room A286, East Lansing, MI 48824, USA; Center for Applied Genetic Technologies, University of Georgia, 111 Riverbend Rd, Room 249, Athens, GA 30602, USA; Department of Plant, Soil and Microbial Sciences, Michigan State University, Plant and Soil Sciences Building, 1066 Bogue Street, Room A286, East Lansing, MI 48824, USA; Center for Applied Genetic Technologies, University of Georgia, 111 Riverbend Rd, Room 249, Athens, GA 30602, USA; Department of Crop & Soil Sciences, University of Georgia, 111 Riverbend Rd, Room 249, Athens, GA 30602, USA; Institute of Plant Breeding, Genetics & Genomics, University of Georgia, 111 Riverbend Road, Room 249, Athens, GA 30602, USA

**Keywords:** *Solanum tuberosum*, self-fertility, genome assembly, genome annotation, nanopore sequencing, *agrobacterium*-mediated transformation, cas9

## Abstract

Availability of readily transformable germplasm, as well as efficient pipelines for gene discovery are notable bottlenecks in the application of genome editing in potato. To study and introduce traits such as resistance against biotic and abiotic factors, tuber quality traits and self-fertility, model germplasm that is amenable to gene editing and regeneration is needed. Cultivated potato is a heterozygous autotetraploid and its genetic redundancy and complexity makes studying gene function challenging. Genome editing is simpler at the diploid level, with fewer allelic variants to consider. A readily transformable diploid potato would be further complemented by genomic resources that could aid in high throughput functional analysis. The heterozygous *Solanum tuberosum* Group Phureja clone 1S1 has a high regeneration rate, self-fertility, desirable tuber traits and is amenable to *Agrobacterium*-mediated transformation. We leveraged its amenability to *Agrobacterium*-mediated transformation to create a Cas9 constitutively expressing line for use in viral vector-based gene editing. To create a contiguous genome assembly, a homozygous doubled monoploid of 1S1 (DM1S1) was sequenced using 44 Gbp of long reads generated from Oxford Nanopore Technologies (ONT), yielding a 736 Mb assembly that encoded 31,145 protein-coding genes. The final assembly for DM1S1 represents a nearly complete genic space, shown by the presence of 99.6% of the genes in the Benchmarking Universal Single Copy Orthologs (BUSCO) set. Variant analysis with Illumina reads from 1S1 was used to deduce its alternate haplotype. These genetic and genomic resources provide a toolkit for applications of genome editing in both basic and applied research of potato.

## Introduction

Cultivated potato, *Solanum tuberosum* Group Tuberosum, (2n = 4x = 48) is the third most consumed food crop in the world (http://www.fao.org/). Potato is a highly heterozygous, clonally propagated, out-crossing autotetraploid with a high genetic load. These factors have led to the highest allelic diversity in a major food crop (Hardigan *et al*., 2017, Hoopes *et al*., 2022). This genetic complexity and redundancy confound functional validation efforts. At the diploid level, forward and reverse genetic strategies can be conducted with more ease (Jansky *et al*. 2016). In addition to genetic complexity, another notable bottleneck is that transformation methods and the recovery of regeneration events are highly variable between genotypes. A transformable and highly regenerable diploid clone that is complemented by an annotated genome assembly would facilitate functional analysis in potato.

The number of emerging model organisms are expanding due to the reduced cost and increased ease of genome sequencing, coupled with advancements in more efficient methods of manipulating genetic material using gene editing. Access to germplasm in which these methods can be readily employed provides a powerful toolkit for efficient functional analysis, simplifying target identification and event characterizations, and increasing the throughput for validating results. A previous study that screened diploid potato germplasm for traits valuable to genetic engineering and gene editing identified several candidate clones (Jayakody *et al*. 2022). 1S1 is a diploid *Solanum tuberosum* Group Phureja clone (hereafter 1S1) that is self-fertile, amenable to genetic transformation, has desirable tuber traits, and could serve as a new model for genetic research in potato (Jayakody *et al*. 2022). Although 1S1 is heterozygous, the availability of a homozygous doubled monoploid of 1S1, DM1S1 facilitates the deduction of the haplotype sequences, providing a valuable resource for future applications of gene editing and genetic engineering to study gene function.

## Methods and materials

### DNA isolation and library preparation

Genomic DNA for Oxford Nanopore Technologies (ONT) sequencing was isolated from greenhouse grown leaves of DM1S1 as described previously (Vaillancourt and Buell 2019). Short sequences were removed using the Circulomics Short Read Eliminator Kit (Circulomics, Baltimore, MD, Cat #SS-100-101-01). Eleven ONT DNA libraries were prepared using the ONT SQK-LSK109 Ligation Sequencing kit and sequenced on six R9 ONT FLO-MIN106 Rev D flow cells. Five of these R9 ONT flow cells were washed and reused according to the Flow Cell Wash kit and protocol (EXP-WSH003, version: WFC_9088_v1_revB_18Sept2019). Sequencing was performed using default settings on an ONT (Oxford, UK) MinION (MinIon 19.12.5 or 19.10.1) using MinKNOW default settings (MinKNOW v3.5.5, v3.6.0, v3.6.5). Details of the sequence generation are provided in Supplementary Table 1.

Genomic DNA for whole-genome shotgun sequencing (WGS) was isolated from young leaves of tissue culture-grown DM1S1 and 1S1 clones using the DNeasy Plant Mini Kit (Qiagen, Hilden, Germany). Illumina TruSeq Nano DNA WGS libraries were prepared and multiplexed using IDT Illumina Unique Dual Index adapters, then sequenced on an Illumina HiSeq 4000 in paired-end mode by the Michigan State University Genomics Core, generating 150 nt reads (Supplementary Table 1).

### RNA isolation and library construction

RNA from leaf and tuber tissue was isolated using a modified hot borate protocol (Wan & Wilkins 1994) and DNase treated using the Ambion Turbo DNA-free Kit (ThermoFisher Scientific, Waltham, MA). Quality RNA, as determined via Qubit, Nanodrop, and gel electrophoresis, was used to isolate mRNA using the Dynabeads mRNA Purification Kit (ThermoFisher Scientific, Waltham, MA, Cat #61011). mRNA was input in the Oxford Nanopore Technologies (ONT) SQK-PCS-109 kit and used to generate full-length cDNA libraries. Resultant libraries were sequenced on ONT R9 FLO-MIN106 Rev D flow cells (Supplementary Table 1).

### Genome assembly

ONT genomic DNA (gDNA) reads were base-called using default parameters with Guppy v3.5.1 (https://community.nanoporetech.com/downloads) using a NVIDIA V100 GPU with the dna_r9.4.1_450bps_hac.cfg configuration file. Reads that passed the base caller quality filter were then filtered to retain reads larger than 10 kb using an awk script (https://github.com/Thilanij/Public/blob/main/10kb_read_filter.awk) yielding a final set of 1,501,797 reads with a total size of 43.9 Gb and ∼52× coverage (Supplementary Table 2).

Contigs were assembled from the final set of ONT reads using Flye v2.4.2 (Kolmogorov *et al*. 2019) with the parameters –nano-raw -g 850 m. The initial assembly was then polished using the final set of ONT reads with two iterations of Racon v1.3.2 (Vaser *et al*. 2017). For each iteration, the reads were mapped to the assembly using minimap2 v2.13 (Li 2018) with the parameter -x map-ont, then polished with the read alignments using Racon with the -u parameter set. The assembly was further polished using the final set of ONT reads with one round of Medaka v.0.12.1. (https://community.nanoporetech.com/downloads). Final polishing was performed with Illumina WGS reads using three rounds of Pilon v1.23 (Walker *et al*. 2014). The Illumina reads were processed by Cutadapt v2.1 (Martin 2011) to remove adapters and to trim low-quality regions with the parameters -n 3 -m 100 -q 30,30. For each iteration, the cleaned reads were aligned to the assembly using Bowtie2 v2.3.4.2 (Langmead and Salzberg 2012) and the alignments sorted with SAMtools v1.10 (Li *et al*. 2009). Pilon was run using the following parameters –fix all –changes –frags. Contigs were scaffolded using a reference-guided approach with Ragtag v1.0.2 (Alonge et al. 2022) using default parameters and the DM v6.1 assembly as the reference (Pham *et al*. 2020). Benchmarking Universal Single Copy Orthologs (BUSCO) v5.2.2 (Simão *et al*. 2015) was used to assess the quality of the final assembly with the orthologs from the EmbryophytaDB V10 data set (*n* = 1614). To assess completeness of assembly in relation to the DMv6.1 reference genome, high confidence v6.1 gene models were aligned to DM1S1 using Minimap2 v2.13 in splice aware mode. Dot plots to visualize chromosome alignments were created using D-Genies (Cabanettes and Klopp 2018).

### Genome annotation

The final genome assembly was repeat masked with RepeatMasker v4.1.0 (Flynn *et al.* 2020) using the DM v6.1 custom repeat library (Pham *et al.* 2020) with the parameters: -s -nolow -no_is -gff. Ab initio gene predictions were made using Augustus v3.3.3 (Stanke *et al.* 2006) with the DM v6.1 training matrix and the softmasked assembly. The Oxford Nanopore cDNA reads for each library were processed with Pychopper v2.4.0 (https://github.com/nanoporetech/pychopper) and aligned to the assembly using Minimap2 v2.17-r941 (Li 2018) using the parameters: -ax splice -uf -G 5000. The cDNA alignments were assembled into transcript assemblies using Stringtie v 2.1.4 (Kovaka *et al.* 2019) with the parameters: -L -m 500. The ab initio gene predictions were refined using two rounds of PASA2 v2.4.1 (Haas *et al.* 2003, Campbell *et al.* 2006) using the transcript assemblies for each cDNA library yielding the set of working gene models. The identification of high confidence gene models and the assignment of functional annotation was performed as described in Pham *et al.* (2020).

### Variant calling

Whole-genome shotgun reads for 1S1 were cleaned using Cutadapt v2.1 to trim low-quality regions using a minimum base quality of 35 and a minimum read length of 50 bp after trimming. Picard v2.18.27 (Picard Tools) was used to convert cleaned fastq reads into an unmapped BAM using FastqtoSam and adapter sequences were marked using Mark Illumina Adapter and SamToFastq, with CLIPPING_ATTRIBUTE = XT and CLIPPING_ACTION = 2. Genomic reads were mapped to the DM1S1 assembly in paired-end mode, flagging secondary hits (-M), using BWA-MEM v0.7.17 (Li, 2013), and then filtered to only keep properly paired reads using SAMtools’ v1.7 view command. MergeBamAlignment was used to restore and adjust metadata as well as allow for any number of insertion or deletion mutations by setting MAX_INSERTIONS_OR_DELETIONS = -1. Duplicate reads were marked using Picard's MARKDuplicates. Reads surrounding insertion/deletions were identified and realigned using GATK's v3.8.1 (McKenna *et al*. 2010) RealignerTargetCreator and IndelRealigner, respectively. Strelka2 v2.9.10 (Kim *et al*., 2018), GATK Haplotypecaller v4.1.4.1 (McKenna *et al*., 2010) and Freebayes v1.3.2 (Garrison and Marth, 2012) were used to call variants. Strelka2 was run on default parameters for the germline configuration, and variants that did not pass the following thresholds set by Strelka2 were removed: IndelConflict, SiteConflict, LowGQX, HighDPFRatio, HighSNVB, and HighDepth. Haplotypecaller was run using the –min-base-quality-score 20 parameter. Freebayes was run using the following parameters, -C 4, –min-mapping-quality 30, –min-base-quality 30. All variants were hard filtered to remove multiallelic sites, and only calls for the alternate allele were kept. These variants were used to create consensus fasta sequences with the DM1S1 assembly using bcftools v1.9.64 (Danecek *et al.* 2021). Ideograms and upset plot were created in R version 4.2.0 using the packages chromPlot (Oróstica and Verdugo, 2016) and UpSetR (Conway *et al*. 2017), respectively.

### *Agrobacterium-*mediated transformation and event characterization

The pHSE401 binary vector containing the HygR gene for hygromycin selection as described by Xing *et al*. (2014) was electroporated into *Agrobacterium tumefaciens* strain GV3101 pMP90 (Koncz *et al*. 1994). *Agrobacterium*-mediated transformation was performed using leaf and internode explants from four-week-old tissue culture plants as described previously (Supplementary Table 3) (Li *et al*. 1999). Briefly, explants were pre-cultured on a Step I media for 2 days then inoculated with *Agrobacterium*. Two days post-inoculation, explants were rinsed with sterile distilled water containing 250 mg/l cefotaxime and 300 mg/l timentin and placed onto Step II media containing 250 mg/l cefotaxime and 300 mg/l timentin for 1 week. Subsequent transfers were placed onto Step II media containing 250 mg/l cefotaxime, 300 mg/l timentin, and 10 mg/l hygromycin. Explants were transferred to fresh Step II media every week. Transformation events (T0 lines) were selected from Step II media and transferred to MS medium supplemented with 250 mg/l cefotaxime, 300 mg/l timentin and 20 mg/l of hygromycin for rooting and selection. Chloroplast counting from 20 guard cells per event was performed to screen for events with altered ploidy according to Ordonez (2014).

DNA from transformation events was isolated from young leaves using the DNeasy Plant Mini Kit (Qiagen, Hilden, Germany). PCR for screening T-DNA insertion was carried out using the GoTaq DNA polymerase (Promega, Fitchburg, WI, United States) using primers designed to amplify an 853 bp region of Cas9 (FWD: 5′ CTCACAAAGGCTGAGAGGGG, RVS: 5′ CCTCCAGGAAATCGATCGGG) with the following thermocycler conditions: one cycle of initial denaturation for 3 min at 95°C, followed by 34 cycles for 30 s at 95°C, 45 s at 60°C and 1 min 30 s at 72°C and a final extension of 5 min at 72°C. Amplicons were visualized on 1.5% (w/v) agarose gels stained with 1X Invitrogen Scientific SYBR-Safe.

Total RNA was isolated using the RNeasy Plant Mini Kit (Qiagen, Hilden, Germany) and DNase treated using the TURBO DNA-free kit (Thermo Fisher, Carlsbad, CA, United States) following manufacturer's instructions. Reverse transcription polymerase chain reaction (RT-PCR) was carried out using the Super-Script III One-Step RT-PCR kit (Thermo Fisher Scientific, Carlsbad, CA, United States). Primers designed to amplify the 853 bp region of Cas9 were also used to confirm expression of Cas9 and as a housekeeping control for RNA quality and DNA contamination, an intron spanning junction of *Actin-11 (*FWD: 5′ AGTGGTCGTACCACCGGTATTGTG, RVS: 5′ ATGATCAGTGAGGTCACGACCTGC) with an expected band size of 131 bp were used for the RT-PCR reactions.

### Assessment of self-fertility

Three clones of four-week-old in vitro 1S1 plants were planted in 19 L plastic pots with a peat and perlite growth medium mixture (Bacto Professional Planting Mix) and placed in a greenhouse with a light intensity of 900–1,000 μmoles s^−1^, 16/8-h light/dark photoperiod. Self-pollinations were conducted from 2022-1-21 to 2022-4-26. Self-fertility was measured as number of viable fruits and number of seeds per fruit after self-pollinating flowers about 2 days pre- and about 1 day post-anthesis. Fruit set was compared using Fisher's exact test for count data conducted in R version 4.2.0.

## Results and discussion

### 1S1 morphology description

**Fig. 1.**
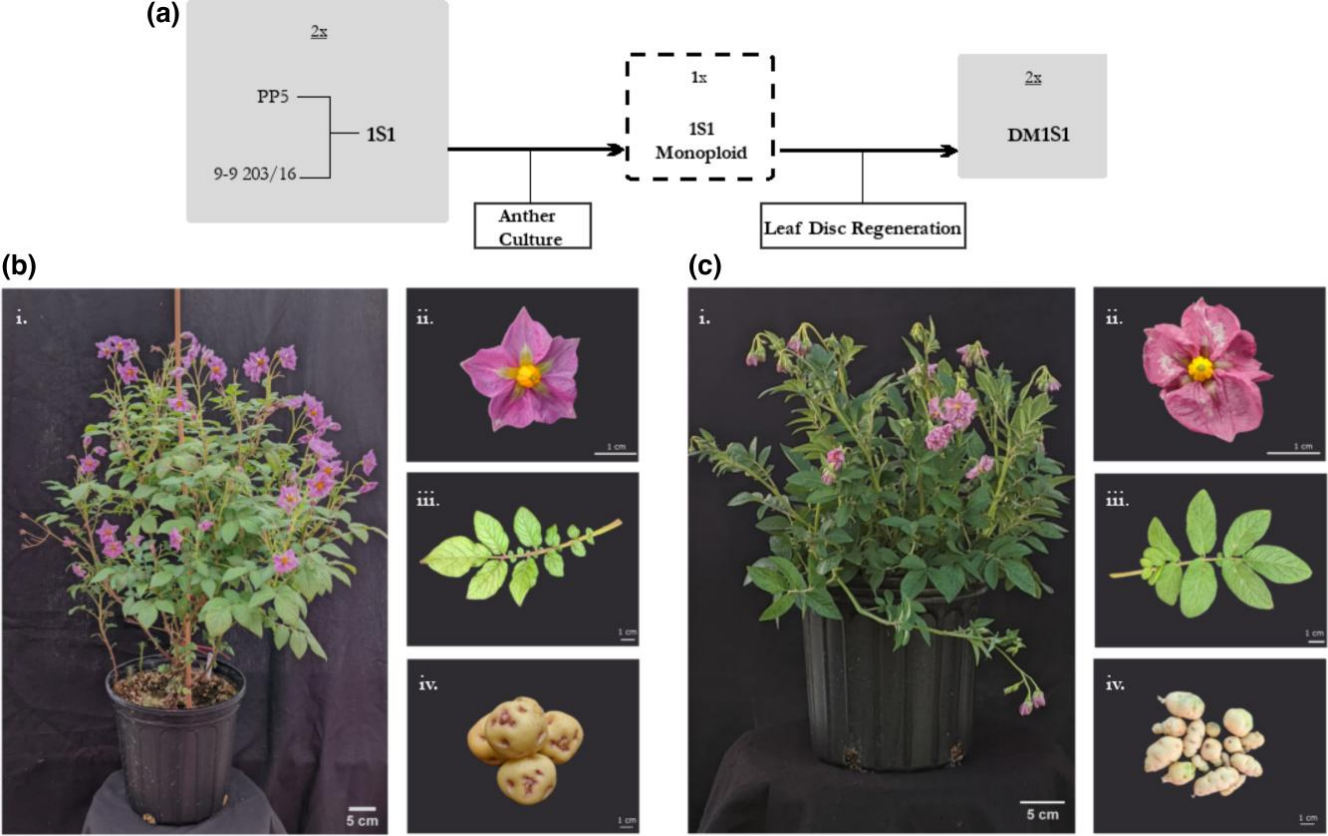
DM1S1 and 1S1 pedigree (a) and morphology for 1S1 (b) and DM1S1 (c) including whole plant (i), flower (ii), leaf (iii) and tuber (iv).

1S1 is a heterozygous *S. tuberosum* Group Phureja diploid potato clone derived from a cross between the doubled monoploid 9–9 203/16 and a heterozygous diploid PP5 generated at Virginia Tech University (Fig. 1a) (Jayakody *et al*., 2022). Although 1S1 is heterozygous, there is currently no potato germplasm to our knowledge that is homozygous as well as being highly regenerable, self-fertile, and having quality tuber traits. To address challenges that arise from heterozygosity, the generation of a haplotype deduced assembly, along with high confidence SNPs that discern the two haplotypes, will facilitate identifying sequences or segregants that could otherwise be challenging to deduce. 1S1 grows vigorously in standard greenhouse conditions and can easily produce hundreds of flowers with male and female fertility. Although capable of producing fruit upon self-pollination, stylar barriers have been observed preventing robust self-fertility (Jayakody *et al.* 2022). 1S1 produces round tubers with red eyes, typical of germplasm found in Group Phureja (Fig. 1b). Under standard tissue culture conditions 1S1 produces long internodes with thick stems, and healthy leaf explants within 3 weeks of culture. 1S1 is amenable to several tissue culture techniques to adjust ploidy such as haploidization through anther culture, which was used in creating DM1S1, and chromosome doubling with 2,4-D, which has been observed at a low frequency in events that have passed through standard regeneration protocol (Supplementary Table 4). Protoplasts from 1S1 have been used in transient assays to assess activity of gene editing reagents (data not shown). Although regeneration from protoplasts have not been tested, 1S1 is regenerable from leaf and internode explants (Jayakody *et al.* 2022). 1S1 is amenable to *Agrobacterium*-mediated transformation but displayed resistance to antibiotic kanamycin when used for plant selection (Jayakody *et al.* 2022).

### Cas9 expressing transgenic events

Challenges with efficient gene editing in plants are limited both by transformation and editing efficiency. In potato, this can be remediated by working at the diploid level, where there are fewer copies to target, but this can be further leveraged by using alternative methods of plant transformation such as viral vector-based gene editing that has shown increased heritable editing efficiencies even when targeting multiple sites at once (Ellison *et al*. 2020). In this approach, a positive strand RNA virus is used to deliver sgRNAs that are tagged with mobile elements that promote germline mutations. The virus with its cargo is then introduced into plant cells through *Agrobacterium* infiltration. Cargo capacity of many viral vectors is a limiting factor for the direct introduction of Cas9, so several methods rely on transgenic Cas9 expressing plants that are then infected with RNA viruses expressing the sgRNAs (Nadakuduti and Enciso-Rodríguez 2021).

To build a model system for rapid functional analysis in potato, the pHSE401 binary vector containing Cas9 driven by a constitutive CaMV 35S promoter was chosen for *Agrobacterium-*mediated transformation using leaf and internode explants. 1S1 has displayed resistance to kanamycin resulting in a high number of escapes, masking its true transformation efficiency (Jayakody et al., 2022) yet alternative selection, such as hygromycin or G418 could result in a lower frequency of escapes. Therefore, we chose a binary vector that also contained hygromycin resistance as the selectable marker. Our results show a transformation efficiency of 29% from leaf explants and 0% from internode explants with 12 putative events collected (Table 1). Of twelve transgenic events, all events maintained their ploidy through tissue culture, except for one which doubled in ploidy (Supplementary Table 4). Expression of Cas9 was confirmed through RT-PCR in all 12 lines (Supplementary Fig. 1). The availability of a Cas9 expressing transgenic self-fertile diploid potato provides a valuable resource for RNA viral vector editing.

**Table 1.** Transformation efficiency of 1S1 using pHSE401 (hygromycin selection).

Genotype	Explant	Number of explants	Explants shooting (%)*^a^*	Rooting Shoots*^b^*	Number of PCR positive events
1S1	Internode	101	0.99%	0	N/A
	Leaf	42	35.71%	12	12

N/A = no events could be collected for explants that did not shoot or for shoots that didn’t root in selection.

*%* explants forming at least one shoot after 4 weeks of culture on Step II media.

*N*umber of shoots collected that rooted in selection media.

### 1S1 self-fertility

**Fig. 2.**
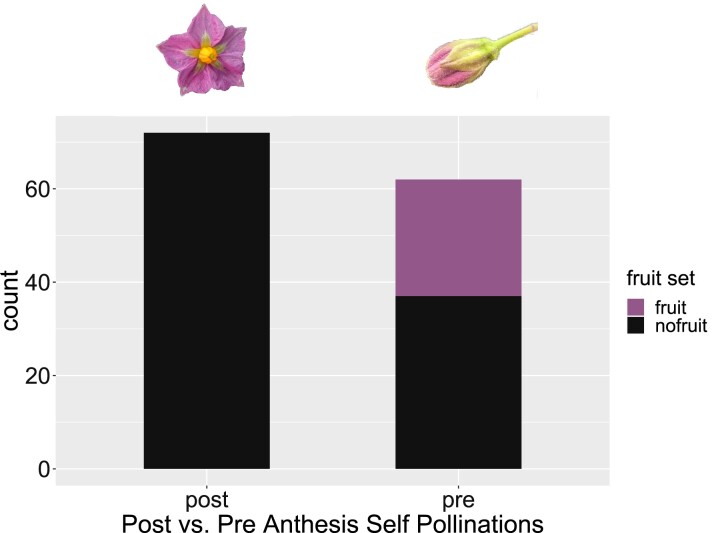
Self-pollinations of 1S1 flowers pre-anthesis resulted in significantly higher viable fruit set (fisher's exact test, *P*-value = 1.656e-10).

Although 1S1 is male and female fertile, stylar barriers are hypothesized to prevent robust self-fertility, which is a valued trait for segregating the T-DNA insertion and fixing of heritable edits. Across several Solanaceae species that experience homomorphic self-incompatibilities it has been observed that factors controlling stylar barriers have reduced activity pre-anthesis, providing opportunities to bypass the gametophytic self-incompatibility system (GSI) (Pandey 1963, Carraro *et al*. 1989, Nettancourt 1997). To investigate conditions for optimal self-fertility, viable fruit set from self-pollinations of pre- and post-anthesis flowers was conducted. There is a significant difference in viable fruit set between self-pollination of pre- and post-anthesis flowers, with 40% of pre-anthesis flowers yielding viable fruit compared with 0% of post-anthesis flowers (Fig. 2, *P*-value = 1.656e-10). For the pollinations that did result in viable fruit, which only occurred in pre-anthesis self-pollinations, the average number of seeds per fruit was 26. Although this overcomes the initial challenge of GSI, a future effort will be to produce a heritable form of self-compatibility by knocking out the S-RNase which is involved in the GSI response in potato (Ye *et al*., 2018, Enciso-Rodriguez *et al.*, 2019).

### DM1S1 morphology description

DM1S1 is a homozygous doubled monoploid derived by chromosome doubling through leaf disc regeneration from an anther-culture-derived monoploid of 1S1 generated at Virginia Tech University (Fig. 1a). DM1S1 grows vigorously for a doubled monoploid in standard greenhouse conditions. It can produce tubers, although without red eyes, and lower yielding than 1S1 with an average tuber number of 9 and an average individual tuber weight of 3.4 grams in the greenhouse (Fig. 1c). DM1S1 is limited by its low regeneration rate and poor male fertility (Supplementary Fig. 2). Although DM1S1 produces flowers that are female fertile, no viable fruit or seed is observed when used as a pollen parent.

### Assembly and annotation of the DM1S1 genome

A total of 44 Gbp of ONT gDNA reads with an N50 read length of 35,482 bp was used with Flye to assemble the DM1S1 genome. The final polished contig set is composed of 299 contigs with a total size of 735.7 Mb, N50 contig size of 11.6 Mb and a maximum contig length of 47.5 Mb (Table 2). Reference-based scaffolding using Ragtag with DM v6.1 inverted a contig on chromosome 4 near the predicted centromere. Alignments to other structurally validated potato assemblies supported the manual correction of this inversion (Supplementary Fig. 3). The completeness of the DM1S1 assembly was determined by assessing the presence of conserved plant orthologs as well as species specific gene models present in the potato reference genome. The final polished assembly represents a nearly complete genic space, shown by presence of 99.6% [C:99.5% (S:97.8%, D:1.7%), F:0.1%, M:0.4%, n:1614] of the BUSCO plant orthologs and 99.83% of DM v6.1 high confidence gene models (Table 2). Whole-genome shotgun Illumina reads which were used for polishing were realigned to the final scaffolded assembly and the frequency of mismatches per base mapped was calculated as 0.24%, indicating a low frequency of errors in the final assembly.

**Table 2.** Genome assembly metrics.

	DM1S1 final polished assembly
BUSCO/V5	C:99.5%[S:97.8%,D:1.7%],F:0.1%,M:0.4%,n:1614
Contig	
ȃTotal length (bp)	735,705,034
ȃNumber	299
ȃN50 length (bp)	11,625,267
ȃLongest contig (bp)	47,526,794
Chromosome scale	
ȃTotal length (bp)	735,746,890
ȃNumber	13
ȃN50 length (bp)	60,143,786
ȃLongest scaffold (bp)	88,174,907
Percent of DMv6.1 high confidence gene models aligned	99.83%
Percent of DM1S1 reads mapping back to assembly	99.98%
No. of mismatches/base mapped from (error rate) from DM1S1 reads	0.24%

We used a trained gene finder and empirical transcript evidence to annotate protein-coding genes in DM1S1. The final gene model working set consisted of 52,348 gene models of which, 43,829 gene models are high confidence representing 31,145 protein-coding genes. The working and high confidence models represents 97% [C:91.1% (S:66.2%, D:24.9%), F:5.9%, M:3.0%, n:1614] and 96.6% [C:90.6% (S:65.8%, D:24.8%), F:6.0%, M:3.34%, n:1614] of the BUSCO orthologs, respectively (Table 3).

**Table 3.** Genome annotation metrics.

	Working Set	High Confidence Set
BUSCO/V5	C: 91.1%[S:66.2%,D:24.9%],F:5.9%,M:3.0%,n:1614	C: 90.6% [S:65.8%, D: 24.8%], F:6.0%, M: 3.34%
number of genes represented	39,594	31,145
number of gene models	52,348	43,829
max transcript	17,418	17,148
max CDS	17,148	17,148
max exon	7,929	7,929
max intron	29,908	14,210
avg transcript	1,578	1,772
avg CDS	1,119	1,227
avg exon	297	303
avg intron	616	606
single exon transcripts	14,488	10,544

### Deduction of 1S1 haplotype

**Fig. 3.**
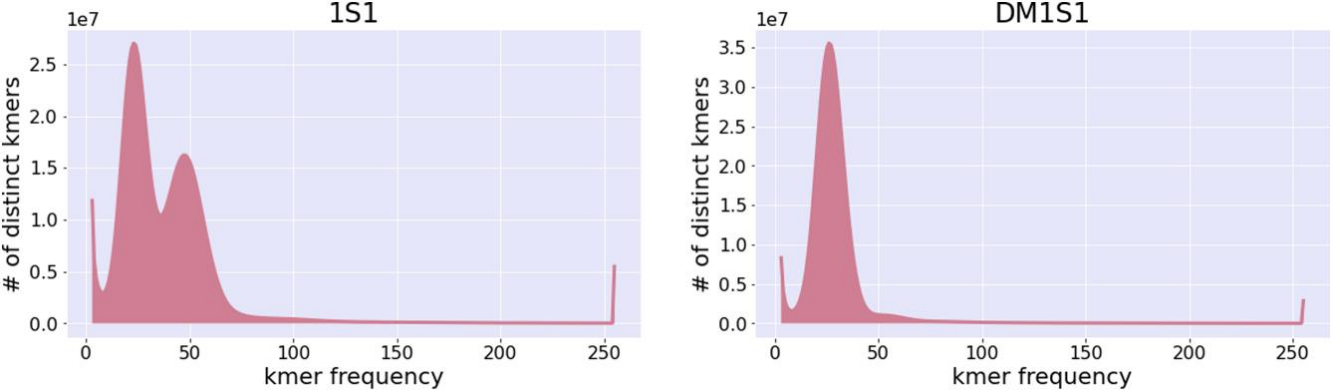
*k*-mer spectra of illumina short read sequences for 1S1 and DM1S1.

**Fig. 4.**
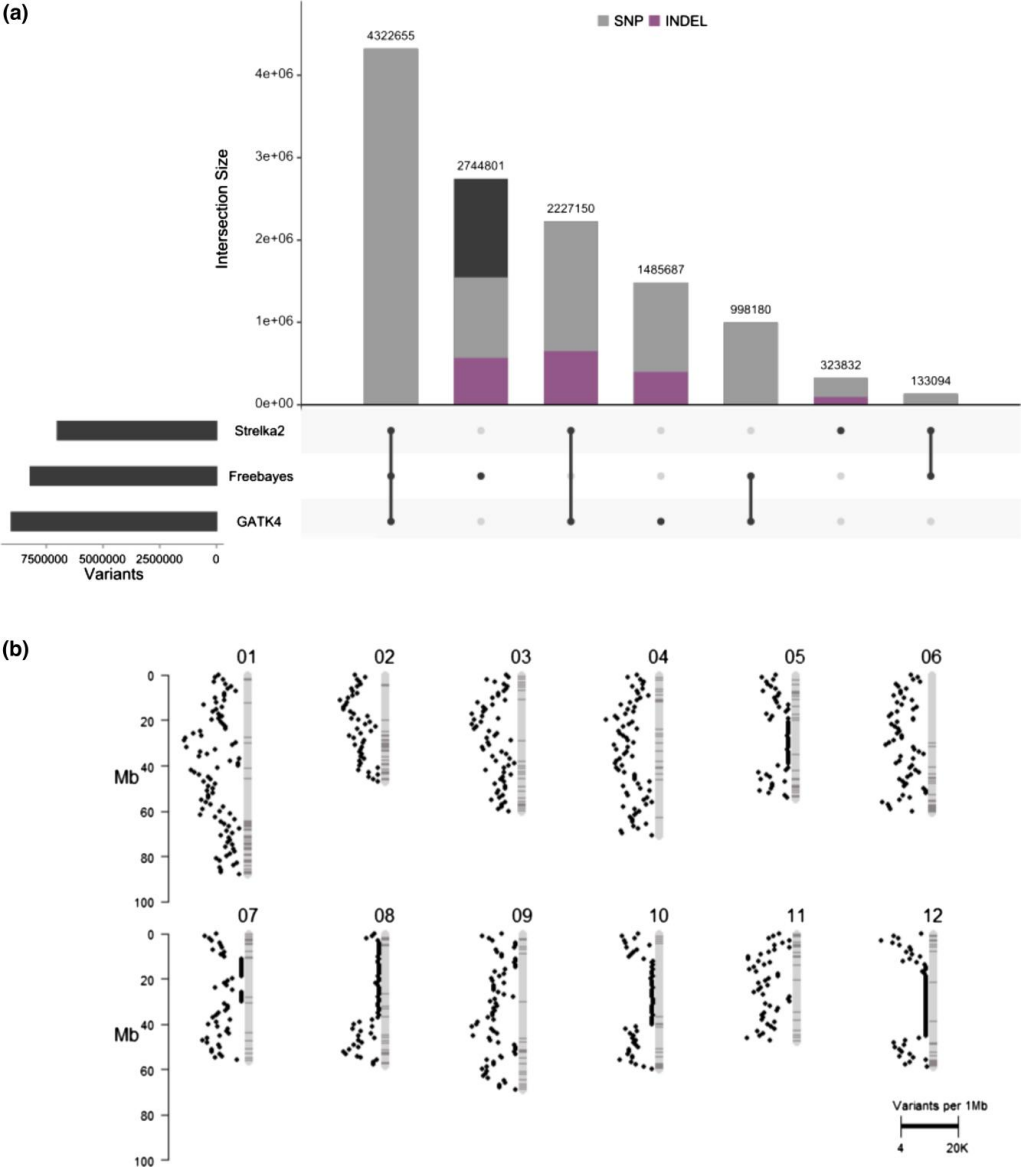
Upset plot displaying variants intersecting between variant calling approaches, where variants not indicated as SNP or INDEL represent complex or multi-nucleotide polymorphisms (a). Ideogram of variants shared among variant calling approaches per 1 Mb, where bands on the ideogram represent the positions of the representative high confidence gene models for DM1S1 (b).

Heterozygosity estimates from Illumina short reads of 1S1 using GenomeScope2.0 (Ranallo-Benavidez *et al*. 2020) with *k* = 31 predict 1.19% intergenomic diversity between haplotypes (Fig. 3). Given the heterozygosity of 1S1, we leveraged the DM1S1 assembly to deduce the alternate haplotype of 1S1. Variants were called using Strelka2, GATK4, and Freebayes, resulting in a total of 7,006,731 variants from Strelka2, 9,033,672 variants from GATK4, and 8,198,730 variants from Freebayes (Fig. 4a). Of those variants, 4,322,655 SNPs were shared among all variant calling approaches (Fig. 4b). To provide a chromosome scale assembly for the deduced alternate haplotype of 1S1, variants from each variant calling approach were combined with the DM1S1 assembly into consensus sequences.

## Supplementary Material

jkad036_Supplementary_Data

## Data Availability

The clones, DM1S1 and 1S1, are available through the United States Department of Agriculture Potato Genebank. The 1S1 constitutively expressed Cas9 lines are available upon request from the Douches’ potato breeding and genetics program at Michigan State University (douchesd@msu.edu, https://www.canr.msu.edu/potatobg/index). The raw genomic sequences and ONT cDNA are available in the NCBI SRA database under BioProject PRJNA888072. The DM1S1 genome assembly and annotation results as well as the 1S1 alternate haplotype assembly are available in the Dryad Digital Repository (doi:10.5061/dryad.5x69p8d70) and on Spud DB (http://spuddb.uga.edu/). Supplementary tables and vector graphic versions for all supplementary figures are available at figshare: https://doi.org/10.25387/g3.21936339. Supplementary Fig. 1 shows PCR and RT-PCR results to confirm T-DNA integration and expression. Supplementary Fig. 2 shows the limitations of DM1S1. Supplementary Fig. 3 shows dot plots from chromosome 4 of DM1S1 compared to structurally validated potato assemblies to validate inversion correction. Supplementary Table 1 describes the sequence datasets generated for this study. Supplementary Table 2 describes the ONT reads used for the assembly. Supplementary Table 3 provides a description of the media used for regeneration. Supplementary Table 4 summarizes the ploidy estimations for the transgenic events.
